# Perceptions of primary and secondary relationships in polyamory

**DOI:** 10.1371/journal.pone.0177841

**Published:** 2017-05-18

**Authors:** Rhonda N. Balzarini, Lorne Campbell, Taylor Kohut, Bjarne M. Holmes, Justin J. Lehmiller, Jennifer J. Harman, Nicole Atkins

**Affiliations:** 1 Department of Psychology, University of Western Ontario, London, Ontario, Canada; 2 Department of Psychology, Champlain College, Burlington, Vermont, United States of America; 3 Department of Psychology, Harvard University, Cambridge, Massachusetts, United States of America; 4 Department of Psychology, Colorado State University, Fort Collins, Colorado, United States of America; 5 Department of Psychology, Portland State University, Portland, Oregon, United States of America; Universita Cattolica del Sacro Cuore Sede di Roma, ITALY

## Abstract

In consensually non-monogamous relationships there is an open agreement that one, both, or all individuals involved in a romantic relationship may also have other sexual and/or romantic partners. Research concerning consensual non-monogamy has grown recently but has just begun to determine how relationships amongst partners in consensually non-monogamous arrangements may vary. The current research examines this issue within one type of consensual non-monogamy, specifically polyamory, using a convenience sample of 1,308 self-identified polyamorous individuals who provided responses to various indices of relationship evaluation (e.g. acceptance, secrecy, investment size, satisfaction level, commitment level, relationship communication, and sexual frequency). Measures were compared between perceptions of two concurrent partners within each polyamorous relationship (i.e., primary and secondary partners). Participants reported less stigma as well as more investment, satisfaction, commitment and greater communication about the relationship with primary compared to secondary relationships, but a greater proportion of time on sexual activity with secondary compared to primary relationships. We discuss how these results inform our understanding of the unique costs and rewards of primary-secondary relationships in polyamory and suggest future directions based on these findings.

## Introduction

While monogamy remains the most common romantic relationship arrangement in North America, consensual non-monogamy (CNM) is prominent, with estimates derived from internet samples suggesting that approximately 4–5% of individuals are currently involved in some form of consensually non-monogamous relationship [1], and other research suggesting that approximately one in five people have previously been a part of a CNM relationship at some point during their lifetime [2]. CNM relationships are those in which partners explicitly agree that they or their partners can enter romantic and/or sexual relationships with other people [3, 4]. CNM relationships can take many forms, but the focus of the present research is polyamory, which refers to an identity in which people philosophically agree with and/or practice multi-partner relationships, with the consent of everyone involved [4–7]. Although the term polyamory indicates permission to engage in sexual or romantic relationships with more than one partner, the nature of these relationships and how individuals approach them can vary from one person partnering with multiple people, to members of a couple dating a third (triad), to two couples in a relationship with each other (quad), to networks of people involved with each other in various configurations [8–11].

Polyamory includes many different styles of intimate involvements, however, most polyamorous-identified individuals report having two concurrent partners [12], and one of the most commonly discussed polyamorous relationship configurations is characterized by a distinction between primary and secondary relationships [13–14]. In this configuration, a primary relationship is between two partners who typically share a household (live together) and finances, who are married (if marriage is desired), and/or who have or are raising children together (if children are desired) [9]. Partners beyond the primary relationship are often referred to as non-primary partners or ‘secondary’ partners. A secondary relationship often consists of partners who live in separate households and do not share finances [9]. In general, secondary partners are afforded relatively less time, energy, and priority in a person’s life than are primary partners. Furthermore, a secondary relationship often consists of less ongoing commitments, such as plans for the future [13–14]. It is worth noting that much of differences discussed here have been speculated to exist, though primarily in non-empirical sources (e.g., popular blogs), and have not been empirically tested.

Primary-secondary relationships can occur through circumstance (e.g. an individual has been in a relationship with one partner and has developed greater interdependence with that partner than others), or through conscious choice (e.g. a commitment to hold the primary relationship as more significant, or to prioritize the primary relationship over other relationships;) [13–14]. Importantly, not all polyamorists have primary relationships with additional secondary partners, and some polyamorists categorically reject the hierarchical distinctions implied by primary-secondary relationships [8]. Although much has been said and written about the primary-secondary distinction in polyamory, very little of it has come from empirical research. As such, research is needed to determine whether our most basic assumptions about these relationships hold true. For example, are there indeed reliable differences between primary and secondary relationships, such that those who identify a partner to be primary are in fact more likely to live with this partner and to report greater relationship duration with that partner? Beyond this, we also seek to assess whether reliable differences emerge on important relationship outcomes, such as commitment, communication, and sexual frequency. Due to the mixed feelings towards primary-secondary relationships within the polyamory community [8], and vast differences in relationship configuration, we therefore limited our sample to polyamorous individuals who personally identified one partner to be primary and another partner to be non-primary.

### Previous research on CNM and goals of current research

The emphasis on romantic and sexual commitments distinguishes polyamory from other types of consensual non-monogamy, such as swinging [15–16] or “open” relationships [17–18]–relationships in which partners agree on sexual relations with others, either as a couple or independently, but operate with minimal emotional and romantic capacity [4–5]. Despite this distinction, most research exploring polyamory collapses polyamory under the broad category of CNM with these other relationship types (though it is important to note that forming committed relationships with multiple partners is quite distinct from having fleeting relationships or casual sex partners on the side). Research shows that individuals in CNM relationships are as equally satisfied with and committed to their relationships as individuals in monogamous relationships [4]. Additionally, consensually non-monogamous and monogamous couples do not differ in reports of relationship quality (e.g., satisfaction, sexual frequency, jealousy, longevity) or psychological well-being (e.g., happiness, depression) [5]. These studies, therefore, suggest that CNM relationships do not significantly differ from monogamous relationships on a number of relationship quality indicators. However, as polyamory involves more intimate involvements than other forms of CNM, meaningful relationship processes may extend to partners beyond the initial dyad, a similarity that may not be expected in open relationships or swinging. More specifically, in open relationships or swinging arrangements, we would not expect substantial commitment or investment to occur with partners beyond the initial dyad because these relationships are typically premised around sex. However, as polyamory extends beyond sexual connection, individuals may report that commitment does exist with partners beyond the initial dyad. Current research is just beginning to explore potential differences in the relationship dynamics an individual has with multiple partners [19]. For example, Mogilski and colleagues [19] found no significant differences between relationship satisfaction ratings of monogamous partners and CNM primary partners, however, the difference between ratings of monogamous partners and CNM secondary partners was marginally significant, such that CNM participants reported higher relationship satisfaction with their primary partner than with their secondary partner. There were some important limitations, however, in their study: the number of individuals with two or more partners was small (e.g. *n* = 76) and the sample involved CNM participants without distinguishing among the different types of CNM. In this case, the authors collapsed across the various forms of non-monogamy (i.e., swinging, open relationships, polyamory) without providing details about how many of these participants fell into each CNM category. Investigating how polyamorous individuals think, feel, and behave within their different romantic relationships is essential for developing an understanding of the psychological processes involved in the maintenance of multiple simultaneous romantic relationships.

#### Relationship acceptance and secrecy

Approximately 25.8% of individuals who practice polyamory have experienced discrimination [20–21]. While previous research has highlighted the fact that polyamory is not widely accepted and is a socially stigmatized relationship configuration [22], to our knowledge no research has empirically tested whether individuals with more than one romantic partner perceive a lack of acceptance from family and friends, and further, whether this acceptance varies across relationships.

One important source of relationship acceptance is the family [23]. Because polyamory challenges the monogamous “ideal” relationship, polyamorists may recognize that sanctions exist for those who do not comply with these conventions. More specifically, Goffman [24–25] suggests that in an attempt to maintain compatibility between personal and social identities, individuals who are subject to stigma may employ strategies to reduce the possibility that others will notice their involvement in discredited behavior [26]. This task is accomplished by *passing*, or the “management of undisclosed discrediting information about [the] self” [24], and by *covering*, which is the “effort to keep the stigma from looming large” [26]. Because primary relationships are more likely to be partnerships in which the couple has been together for a longer period of time, are more likely to be married, and more likely to live together, it is conceivable that these relationships could be more likely to *pass* for monogamous partnerships or *cover* an individual’s polyamorous identity than secondary relationships, providing one potential reason for more acceptance from family for primary relationships. We hypothesized that in polyamorous relationships, the mean amount of perceived acceptance from family for primary relationships would be greater than the mean amount of acceptance for secondary relationships (Hypothesis 1).

Additionally, it is likely that the expectations from important peers (e.g., friends) lean towards cultural monogamy norms given their pervasiveness [27]. We therefore hypothesized that the mean amount of perceived acceptance from friends for primary relationships would also be greater than the mean amount of acceptance for secondary relationships (Hypothesis 2). While we expect primary relationships to receive greater acceptance from family and friends, contrary to family, individuals can select their friends and may be likely to select friends who are either similar to or more accepting of their relationships. We thus predicted that family would be perceived as less accepting of secondary relationships than friends (Hypothesis 3).

Furthermore, the desire to comply with customs and norms, or to avoid stigma, could result in greater secrecy about polyamorous relationships, particularly, when it comes to relationship partners beyond the primary relationship members. We therefore hypothesized that in polyamorous relationships, the mean amount of romantic secrecy would be greater for secondary relationships than the mean amount of romantic secrecy reported for primary relationships (Hypothesis 4). While stigma towards CNM has been documented at the general level (i.e., that people typically favor monogamy), no research to this point has assessed how polyamorous individuals experience stigma in their relationships, and whether acceptance and secrecy was experienced in all relationships, or in fact predicted by the status of the relationship (i.e., whether one is primary or secondary).

#### Relationship investment and commitment processes

Interdependence theory posits that individuals initiate and maintain relationships because of the benefits of interactions in a relationship [28–30]. As relationships develop, the interaction amongst partners yields outcomes in the forms of rewards (e.g. sexual pleasure, relationship satisfaction, security), and costs (e.g. increased responsibility, distress or anxiety, despair, fear) [31]. Rusbult’s Investment Model [32–33], based on Interdependence Theory, proposes that motivation to maintain a relationship is the product of four variables: (1) *investment size*, or the direct and indirect resources (e.g., time invested, cognitive interdependence, plans for the future) that represent the ways one is bound to the relationship; (2) *satisfaction*, or how rewarding the relationship is; (3) *quality of alternatives*, or the degree to which one believes that one’s needs could be fulfilled in another relationship; and (4) *commitment*, or the subjective representation of dependency, experienced as a feeling of psychological attachment to the partner and desire to maintain the relationship [31]. Relationship commitment typically arises when one is highly invested and satisfied, and perceives that there are no better options to one’s current relationship. Commitment, in turn, promotes relationship persistence.

In polyamorous relationships, anecdotal evidence suggests primary partners may afford certain rewards because primary partners can share in major life decisions and can help to promote greater levels of interdependence (e.g., joint finances, cohabitate, etc.) [8]. Some experiences and behaviors that are more common among primary partnerships, such as relationship approval and the ability to exist as a publicly recognized couple (especially when secrecy in other relationships is salient) may be additionally rewarding. In contrast, other experiences and behaviors that are likely more common among secondary relationships may have relationship deterring effects, such as maintaining a romantic bond in social climates that marginalize and devalue polyamorous relationships. For these reasons, we further expected that it should be more difficult to develop interdependence in secondary relationships compared to primary relationships.

A practical matter to also consider is the degree to which one invests in and is therefore able to commit to a relationship, given that many investments are, by their nature, limited. More specifically, if the primary partner is the recipient of many of the investments typical in traditional relationship trajectories (moving in together, getting married, having children, etc.), there are simply fewer resources left to invest into relationships with secondary partners, and thus, fewer opportunities to become truly interdependent. Additionally, previous research utilizing the Investment Model Scale found that individuals in marginalized relationships invest significantly less than individuals in nonmarginalized relationships [34]. Taken together, we predicted that the mean amount of investments for primary relationships would be greater than the mean amount of investments reported in secondary relationships (Hypothesis 5).

Additionally, it has been suggested that denying or hiding a relationship can decrease relationship satisfaction because it can represent a devaluing of the relationship [35], and creates anxiety about the relationship itself [36]. Keeping a relationship secret is also linked to elevated reports of physical and psychological stress [37], another factor that might be expected to lower relationship quality. Recent research has also found that within CNM relationships, participants reported higher overall relationship satisfaction with primary compared to secondary relationships and considered their primary partner to be more desirable as a long-term mate than their secondary partner [19]. Thus, we predicted that individuals in polyamorous relationships would be more satisfied with primary relationships than secondary relationships (Hypothesis 6). That said, to the degree that individuals have chosen to stay with a primary partner while pursuing other alternatives (as opposed to leaving that relationship entirely), we predicted that the perceived quality of alternatives would be lower for assessments of primary compared to secondary relationships (Hypothesis 7). More specifically, individuals in polyamorous relationships should be less likely to desire leaving the primary partner for another equivalent relationship, and somewhat more likely to desire leaving a secondary partner for another equivalent relationship. Lastly, to the extent that the above predictions are true—that primary relationships are indeed associated with greater satisfaction and investments and fewer alternatives—this would be expected to translate to greater commitment for primary compared to secondary relationships, consistent with the central prediction of the Investment Model (Hypothesis 8). Additional reasoning for this hypothesis comes from other research finding that marginalization is a negative predictor of commitment [34]. Given that secondary relationships are thought to be more marginalized than primary relationships, we would expect commitment to the former to be lower than commitment to the latter.

#### Relationship communication

Communication is an extremely valuable skill in any relationship, but particular importance is placed on communication in the context of polyamorous and other CNM relationships. Polyamorists actively sustain their engagements with multiple partners through an ideology that emphasizes open and honest communication [8]. To facilitate this communication, most individuals practicing polyamory report making agreements, or freely chosen rules with their partners regarding intimate behaviors, preferred level of knowledge about other partners, and so forth [9, 12]. Agreements are particularly salient and important to sustaining primary relationships in polyamory for multiple reasons. In order to make agreements that facilitate other relationships while protecting the primary relationship, communication amongst partners about their relationship, needs, and expectations is essential. In previous research, communication was found to be one of the variables that contributed to maintaining commitment between primaries in long-term polyamorous relationships [38]. Thus, we hypothesized that the level of communication about the relationship would be perceived as greater in primary relationships than secondary relationships (Hypothesis 9). Further, we expected that when asked to compare their relationships to most other people participants know, the quality of communication would be perceived as greater for primary relationships than secondary relationships (Hypothesis 10). This may, in part, be due to a greater need to communicate, and due to more practice communicating, considering that primary relationships tend to have greater relationship duration (to be discussed in more detail in the Results).

#### Percentage of time spent on sexual activity

While most of the predictions discussed thus far highlight the potential rewards attributed to primary relationships in comparison to secondary relationships, one potential reward that can be attributed to secondary relationships involves sexual activity. Given that secondary relationships tend to be newer partnerships and that the typical trajectory of sexual activity in relationships involves a greater frequency of sex early on that declines over time [39], we predicted that polyamorists would report a greater amount of time spent engaging in sexual activity (out of the total time spent together) in secondary relationships (Hypothesis 11). Importantly, we focus on the percentage instead of the frequency because it is presumed that participants will spend more time in general with primary partners. If people spend less total time with secondary compared to primary partners, than frequency comparisons would be unfairly biased towards less frequent sex with secondary partners by virtue of the lack of access. A percentage/proportion measure controls for the different amount of time primary and secondary partners spend together. In the present research, we test predictions regarding differences in the perceptions of two concurrent romantic relationships (i.e., primary and secondary relationships) of self-identified polyamorous individuals. Specifically, we focus on acceptance and secrecy, investment and commitment processes, as well as communication about the relationship and sexual frequency across relationships.

## Materials and methods

### Participants

Research was conducted in accordance with the ethical guidelines of the American Psychological Association. Informed consent was received from each participant digitally (each participant indicated they read the consent form and agreed to take part before proceeding with the survey). Additionally, this research was approved by the IRB at Champlain College (Vermont, US). A convenience sample of adults (*N* = 3,530), primarily from the United States (*n* = 2,428), who identified as polyamorous was recruited from various internet forums, dating sites, and Facebook group pages to take part in the study. Most of these websites and groups were specifically geared toward a polyamorous audience (e.g., Facebook groups for Polyamorous individuals, advertisements in polyamorous blogs). Participants were informed that in order to participate in the study, they must identify as polyamorous, be at least 18 years of age, and currently be in a relationship with at least one person. Prospective participants were provided a link (see: https://harvard.az1.qualtrics.com/SE/?SID=SV_bJhORcv4yrHTcA5) that re-directed them to a survey hosted on Qualtrics.

Most participants reported having at least two partners (72.8%; *n* = 2,571) at the time of testing, however, we only collected detailed information on up to two partners due to time constraints and concerns about participant burden. As the focus of the current study is assessing differences between primary and secondary relationships, we limited participants in the current study only to those who indicated that the first person listed was a primary partner, and the second person listed was a non-primary partner (37.05% of the full sample; *n* = 1308). Within this sub-sample, the majority (58.6%) of respondents identified as female (*n* = 766), 36.8% identified as male (*n* = 481), 1.0% identified as transgender (*n* = 13), 3.5% identified as another gender (*n* = 46), and 0.20% were missing responses (*n* = 2). Of the people who wrote in their own gender identity, common examples included “trans-gendered,” “non-gendered,” “gender-queer,” “co-gendered,” “non-binary,” and “gender-fluid.” With respect to sexual orientation, most (51.2%) respondents identified as bi- or pansexual (*n* = 667), 39.0% identified as heterosexual (*n* = 510), 2.8% identified as lesbian or gay (*n* = 36), 7.0% identified as other (*n* = 92), and 0.2% were missing responses (*n* = 3). Participants who identified their sexual orientation as “other” were allowed to write in their identity; common responses were “hetero-flexible,” “fluid,” “queer,” “bi-curious,” “polysexual,” and “asexual.” The age of participants ranged from 18 to 78 years old, and the average age was 35.26 (*SD* = 10.45).

### Procedures

For the purpose of this study, polyamory was defined as “the practice or acceptance of having multiple simultaneous romantic relationships where everyone involved consents” for the participants. Data were collected as part of an online testing session between December 2012 and January 2013. Participants answered a battery of questionnaires, including demographic questions about themselves and all partners they had, as well as detailed questions about their relationship experiences with a primary and a single secondary partner only. Questions addressed concepts including jealousy, communication, satisfaction, quality of alternatives, investment-size, commitment, sex, secrecy, and perceived approval.

### Measures

#### The concept of a primary-secondary relationship

Respondents were asked to provide the initials of partners #1 and #2, and then were asked a series of questions about their relationships with these partners. The survey was programmed such that the initials for each partner were piped into the questions to avoid confusion regarding which partner was being asked about. To assess assumptions about primary-secondary partnerships, participants were asked to indicate the number of years and months they had been in a relationship with partner #1 and partner #2. Next, to assess whether partner #1 or partner #2 was considered to be a primary partner, respondents were given five options: 1 = *Yes*, *partner (partner’s initials) is a primary relationship*, 2 = *Yes*, *partner (partner’s initials) is a primary relationship*, *but I also have others that are considered primary*, 3 = *No*, *partner (partner’s initials) is not a primary relationship*, 4 = *No*, *I do not believe in considering one partner primary*, and 5 = *None of the above* (with an option to explain after). Lastly, respondents were asked to indicate whether they lived with partner #1 or partner #2 with the simple response option of *yes* or *no*. These questions were presented within the demographic questions, prior to presenting our primary measures.

With regard to the following measures, participants answered each question for two concurrent relationship partners. In the following discussion of measures, “partner ()” reflects the initials of the persons that each participant indicated as their first and second listed partners.

#### Relationship acceptance and secrecy

A one-item measure (on a 9-point Likert-type scale, anchored 1 = *do not agree at all*, 9 = *agree completely*) assessed relationship acceptance from family (e.g., “My family is accepting of my relationship with partner”); and from friends (e.g., “My friends are accepting of my relationship with partner ()”) [34]. These items were intended to be analyzed separately, as was established in our pre-registered hypotheses and analytic plan, however, we did explore the possibility of using a composite of these items, but due to the poor reliability of these items together (primary partner α = .56; secondary partner α = .59), we did not proceed with the aggregate.

Participants answered two questions (on a 9-point Likert-type scale, anchored 1 = *do not agree at all*, 9 = *agree completely*) regarding experiences with secrecy in their relationship(s). The items used included, “During the past week, my relationship with partner () was secret from someone,” and “During the past week, I hid some things about my involvement with partner () from some people” (primary partner α = .66; secondary partner α = .90) [40].

#### Investment and commitment processes

The measure of investment size contained three items based on the Investment Model Scale (IMS) [41]. Items assess the ways in which people get bound by resources in the relationship and thus the potential costs of losing the relationship (e.g., “I have put a great deal into this relationship that I would lose if the relationship were to end,” “I feel very involved in our relationship–like I have put a great deal into it,” and “Compared to other people I know, I have invested a great deal in my relationship with partner”); (9-point Likert-type scale, anchored 1 = *do not agree at all*, 9 = *agree completely*; primary partner α = .69; secondary partner α = .90).

Participants answered three questions regarding their satisfaction with romantic relationship partners. The items used were based on the IMS [41] and included, “My relationship with partner () is much better than others’ relationships,” “I feel satisfied with our relationship,” and “Our relationship makes me very happy” (on a 9-point Likert-type scale, anchored 1 = *do not agree at all*, 9 = *agree completely*; primary partner α = .82; secondary partner α = .82).

Five questions regarding the perceived quality of alternatives were included. The items used were based on the IMS [41] and included, “My alternatives to our relationship are close to ideal (dating another, spending time with friends or on my own, etc.),” “My alternatives are attractive to me (dating another, spending time with friends or on my own, etc.),” “My needs for intimacy, companionship, etc. could easily be fulfilled in an alternative relationship,” “If I weren't dating partner (), I would do fine–I would find another appealing person to date,” and “The people other than partner () with whom I could become involved are very appealing” (on a 9-point Likert-type scale, anchored 1 = *do not agree at all*, 9 = *agree completely*; primary partner α = .78. secondary partner α = .85).

Participants responded to four questions, based on the IMS [41], about their commitment. The items used included, “I feel very attached to our relationship–very strongly linked to partner (),” “I am oriented toward the long-term future of my relationship (for example, I imagine being with partner () several years from now),” “I intend to stay in this relationship,” and “I am committed to maintaining my relationship with partner ()” (on a 9-point Likert-type scale, anchored 1 = *do not agree at all*, 9 = *agree completely*; primary partner α = .88; secondary partner α = .92).

#### Relationship communication

Communication in the relationship was measured using a 9-point Likert-type scale (anchored 1 = *never*, 9 = *daily*) asking participants to consider, “How often you communicate with partner () on average about the following topics?:” “About the quality of your relationship,” “About what love means to you,” “About your relational desires and needs,” “About your sexual desires/needs,” “About another romantic partner/interest of yours or theirs,” “About commitment and the future,” “About feelings of jealousy,” “About scheduling time for each other,” and “About how your family and/or the outside world view your relationship” (primary partner α = .87; secondary partner α = .90). Participants were asked with one item to evaluate the quality of the communication with their partner in comparison to most people they know. Participants responded on a 5-point Likert-type scale (anchored 1 = *well below average*, 5 = *well above average*).

#### Percentage of time spent on sexual activity

Of the time partners spent together, participants were asked to estimate what percentage of that time was spent on sexual activities, from 0%– 100% [42].

### Analytic strategy

To control for the experiment-wise error rate in hypothesis testing associated with conducting a large number of statistical tests [43], the criteria for statistical significance for our pre-registered hypotheses was corrected by using the Bonferroni method; dividing α = .05 by the number of pair-wise tests (.05 / 11 = .0045). Therefore, the *p*-value used across these analyses was set at *p* < .0045 level rather than the typical *p* < .05 level. The hypotheses and the data analytic plan were pre-registered on the Open Science Framework (OSF) prior to conducting the analyses (see: https://osf.io/bgtuy/). Additionally, all of the data and code required to reproduce the analyses presented below are located on the OSF (https://osf.io/vs574/).

## Results

### The concept of a primary partner

Participants answered the same questions about each of the partners they identified as primary and secondary. Participants reported a significantly longer relationship duration with the primary partner (*M* = 8 years and 4 months, *SD* = 7 years and 6 months) than with the secondary partner (*M* = 2 years and 4 months, *SD* = 3 years and 6 months); *t*(781) = 21.91, *p* < .001, Cohen's *d* = 0.96. Additionally, to assess cohabitation and primary status, McNemar’s test for paired nominal data was used. The test is applied to 2 × 2 contingency tables that have a dichotomous variable with matched pairs of subjects. In our study, one repeated dichotomous variable was living/not living with partner. The matched pairs are responses for each of two partners. The test statistic is a χ^2^ value with one degree of freedom and if it is statistically significant, it suggests that the marginal proportions are different from each other (e.g. Are the proportions of primary partners living with participants equal to the proportion of secondary partners living with participants?). We found that participants were much more likely to share a household with their primary partner (72.21%) than with their secondary partner (0.002%); McNemar χ^2^(1) = 932.02, *p* < 0.001, φ = 0.85. This data pattern supports the notion that primary relationships involve greater relationship duration and are more likely to consist of partners who cohabitate, and thus the data support anecdotal and popular claims about the nature of primary-secondary relationships.

### Tests of main predictions

The data were analyzed in a series of paired-sample *t*-tests to compare participants’ perceptions of their primary and secondary relationships. Results from these analyses are presented in Table 1. All of our predictions were supported. Specifically, participants reported more relationship acceptance by family and friends, greater investment size, higher levels of commitment, more relationship satisfaction, greater communication about the relationship and greater quality of communication for primary compared to secondary relationships. On the other hand, participants reported greater romantic secrecy, higher quality of alternatives, and spending a greater proportion of time on sexual activity with secondary compared to primary relationships. Effect sizes of the mean differences appropriate for repeated measures (i.e., Cohen's *d*) were calculated using the value of the *t*-test, the correlation between the two paired-means, and the total sample size. Effect sizes were moderate to large, with the exception of quality of alternatives, which was relatively small. Effect sizes were not predicted *a priori*, but the large sample size, combined with the predominantly moderate to large effect sizes, suggests that the effects are robust. Results for primary and secondary relationships were consistent with the overall sample as well. To see data, syntax, and output for the analyses involving all participants (e.g., data collapsed such that participants who report co-primaries or no primaries are also included), please see: https://osf.io/ph6up/.

**Table 1:** Descriptive statistics, tests of mean differences, and effect sizes for the primary and secondary relationships on major study variables.

Variable	Primary Relationship		Secondary Relationship		Paired Data			
	*M*	*SD*	*M*	*SD*	*n*^a^	*r*^b^	*t*	*d*
Relationship acceptance: Family	7.95	1.87	4.29	2.45	868	.08	36.40	1.68
Relationship acceptance: Friends	8.45	1.18	6.28	2.25	872	.17	27.20	1.19
Romantic secrecy	1.92	1.81	5.29	3.11	875	.23	-30.89	-1.27
Investment size	7.90	1.24	5.15	2.03	875	.26	39.00	1.60
Relationship satisfaction	7.80	1.30	6.40	1.56	875	.10	21.41	0.97
Quality of alternatives	5.92	1.70	6.44	1.59	874	.55	-10.01	-0.32
Commitment level	8.54	0.94	6.31	1.94	874	.19	33.20	1.39
Relationship communication	5.38	1.45	3.98	1.45	908	.43	27.35	0.97
Quality of communication	4.47	0.78	3.59	0.94	918	.17	23.85	1.01
Percentage of sexual activity	20.74	21.11	37.11	27.48	860	.03	-14.09	-0.70

^a^ The sample size varies across analyses because of missing or incomplete data for one or both partners. The analyses were re-run using the subset of participants who responded to every question included in our primary analyses. The effects are essentially the same. Please see the output in the supplementary materials on the OSF: https://osf.io/gxtcn/.

^b^ *r* = the correlation between scores for primary and secondary relationships.

Next, we compared acceptance of secondary partners from family vs. friends (using a paired-samples *t*-test). Consistent with predictions, participants’ perceptions of acceptance for secondary relationships were greater for friends (*M* = 6.27, *SD* = 2.26) than family (*M* = 4.30, *SD* = 2.45); *t*(865) = 22.78, *p* < .001; *d* = 0.83. For exploratory purposes, we performed the same analyses on participants’ perceptions of acceptance for primary relationships, which revealed the same pattern: acceptance was greater for friends (*M* = 8.45, *SD* = 1.18) than family (*M* = 7.93, *SD* = 1.89); *t*(882) = 8.87, *p* < .001; *d* = 0.32). Although the former analysis was preregistered and the latter was not, we have included both to provide a comparison of acceptance from friends vs. family for both primary *and* secondary partners.

### Exploratory analyses

#### Effects of primary-secondary relationship length differences on main analyses

The reported differences between perceptions of primary and secondary relationships for our primary analyses (see Table 1) could potentially be accounted for by the fact that most primary relationships have existed for a longer period of time than secondary relationships. To test whether differences in relationship length are related to, or can account for, the differences between perceptions of primary and secondary relationships, we conducted a series of linear regression analyses in which the difference between perceptions of the primary and secondary relationships for each dependent variable were regressed on the difference in relationship length between the primary and secondary partners (secondary partner relationship duration subtracted from the primary partner relationship duration). The intercept in this analysis is the estimated value of the outcome variable (i.e., the difference between the two repeated measures) when the value of the predictor variable equals zero. Without centering the relationship length difference variable, zero is a meaningful value as it represents a case where there is no difference in relationship length between primary and secondary relationships (and thus the slope represents how much the difference in the dependent variables changes for every unit change in relationship length difference). Therefore, if the difference in length between relationships completely accounted for the mean differences we report in our primary analyses, the intercept in this analysis would be non-significantly different from zero and the coefficient for the predictor variable would be statistically significant and positive (i.e., when individuals report being with the primary partner longer relative to the secondary partner, they would also report more commitment to the primary relative to the secondary). If, however, the mean difference between the dependent variables still emerges when controlling for the difference in relationship length, it would provide more convincing support for our findings. The results of these analyses are presented in Table 2. In every instance the predicted difference between perceptions of the primary and secondary relationships, estimated by the intercepts in the analyses, remained statistically significant! The effect sizes of these mean differences when controlling for the difference in relationship length is also presented in Table 2. The slope was a significant predictor in 9 of the 10 models. In each instance the significant slope indicated that as the difference in relationship length between the primary and secondary relationship became larger, the mean difference in the dependent variable also became larger (e.g., individuals are more invested to their primary relative to secondary relationship when they have been in their primary relationship longer than the secondary relationship). Variability in relationship length is therefore an important factor in understanding differences in perceptions between primary-secondary relationships, but it does not completely account for these differences.

**Table 2:** Linear regression with relationship length difference predicting differences between primary and secondary relationships on primary analyses.

Variable	Primary-Secondary Difference		Paired Data^a^			
	*Intercept (SE)*	*Slope (SE)*	*n*	*r*^b^	*t*	*d*
Relationshio acceptance: Family	3.07 (0.16)**^c^	0.10 (0.02)**	535	.10	18.75	1.09
Relationship acceptance: Friends	1.73 (0.13)**	0.06 (0.01)**	538	.15	13.49	0.76
Romantic secrecy	-2.68 (0.18)**	-0.10 (0.02)**	539	.27	-15.34	-0.80
Investment size	2.58 (0.11)**	-0.00 (0.01)	539	.27	22.56	1.17
Relationship satisfaction	1.54 (0.10)**	-0.03 (0.01)**	539	.14	14.92	0.84
Quality of alternatives	-0.58 (0.07)**	0.02 (0.01)**	538	.57	-8.40	-0.34
Commitment level	2.23 (0.11)**	-0.02 (0.01)*	539	.23	21.26	1.14
Relationship communication	1.66 (0.08)**	-0.05 (0.01)**	558	.49	21.76	0.93
Quality of communication	1.00 (0.06)**	-0.02 (0.01)**	565	.21	17.32	0.92
Percentage of sexual activity	-12.57 (1.80)**	-0.69 (0.19)**	529	.08	-6.98	-0.41

^a^ Estimated mean comparisons when difference in relationship duration was zero.

^*b*^ *r* = the partial correlation controlling for relationship length difference between scores for primary and secondary relationships.

^c^ **p < .01,

*p < .05.

#### Effects of cohabitation on differences in perceptions of each partner

It is also possible that the reported differences in perceptions between the primary and secondary relationship is accounted for by differences in living arrangements between the primary and secondary partners. To test this possibility, we reran our analyses with the subset of participants who did not live with *either* their primary or secondary partner (*n* = 296). As can be seen in Table 3, all of our pre-registered predictions were still supported. Specifically, even when participants did not live with their primary or secondary partners, participants still reported more relationship acceptance by family and friends, lower romantic secrecy, greater investment size, more relationship satisfaction, lower quality of alternatives, higher levels of commitment, greater communication about the relationship, greater quality of communication, and lower sexual frequency for primary compared to secondary relationships. According to these analyses, cohabitating partially, but not entirely, contributes to the magnitude of the differences in the dependent variables.

**Table 3:** Descriptive statistics, tests of mean differences, and effect sizes for primary and secondary relationships among partners who do not cohabitate.

Variable	Primary Relationship		Secondary Relationship		Paired Data			
	*M*	*SD*	*M*	*SD*	*n*	*r*^a^	*t*	*d*
Relationship acceptance: Family	6.80	2.17	4.62	2.23	186	.25	11.03	0.99
Relationship acceptance: Friends	8.02	1.57	6.38	2.17	189	.37	10.43	0.85
Romantic secrecy	2.86	2.48	4.69	2.96	189	.45	-8.72	-0.67
Investment size	7.17	1.39	4.66	1.91	189	.33	17.53	1.48
Relationship satisfaction	7.73	1.31	6.25	1.57	189	.17	10.90	1.02
Quality of alternatives	6.00	1.53	6.79	1.44	189	.51	-7.32	-0.53
Commitment level	8.18	1.21	5.80	1.96	189	.22	15.84	1.44
Relationship communication	5.19	1.40	3.65	1.27	200	.44	15.29	1.14
Quality of communication	4.52	.74	3.56	0.97	203	.28	13.03	1.10
Percentage of sexual activity	30.02	21.92	40.23	28.05	190	.19	-4.38	-0.40

^a^ *r* = the correlation between scores for primary and secondary relationships.

#### Effects of relationship length difference and cohabitation on differences in perceptions of each partner

To assess the cumulative effect relationship length and cohabitation have on the differences we found in our main analyses, we conducted separate linear regression analyses in which difference scores between each of the main measures were predicted with the difference in relationship length between primary and secondary relationships with the subset of participants not living with either partner. The results of these analyses are presented in Table 4. Significant differences in perceptions of the primary and secondary relationships continued to emerge, suggesting that differences in relationship length in conjunction with cohabitation do not completely account for the predicted effects.

**Table 4:** Linear regression with relationship length difference predicting differences between primary and secondary relationships with partners who do not cohabitate.

Variable	Primary-Secondary Difference		Paired Data			
	*Intercept (SE)*	*Slope (SE)*	*n*	*r*^a^	*t*	*d*
Relationship acceptance: Family	1.84 (0.26)**^b^	0.23 (0.07)**	119	.28	7.15	0.79
Relationship acceptance: Friends	1.61 (0.22)**	0.05 (0.06)	120	.26	7.27	0.81
Romantic secrecy	-1.71 (0.27)**	-0.18 (0.07)*	120	.48	-6.28	-0.59
Investment size	2.16 (0.19)**	0.12 (0.05)**	120	.38	11.57	1.18
Relationship satisfaction	1.42 (0.17)**	-0.00 (0.05)	120	.16	8.17	0.97
Quality of alternatives	-0.78 (0.14)**	0.00 (0.04)	120	.50	-5.41	-0.49
Commitment level	2.07 (0.19)**	0.09 (0.05)	120	.31	11.14	1.20
Relationship communication	1.58 (0.12)**	-0.10 (0.03)**	123	.50	12.75	1.15
Quality of communication	0.93 (0.10)**	-0.00 (0.03)	126	.25	9.69	1.06
Percentage of sexual activity	-8.34 (2.68)**	-2.91 (0.71)**	120	.34	-3.11	-0.33

^a^ *r* = the partial correlation controlling for relationship length difference between scores for primary and secondary relationships.

^b^ **p < .01,

*p < .05.

#### The links between investment, relationship satisfaction, and quality of alternatives with commitment for each partner

To test whether investment, relationship satisfaction, and quality of alternatives predict commitment for primary and secondary partners, we conducted a path analysis using the lavaan [44] package in R. In the model, we tested both the within partner and between partner associations. The trio of predictor variables were set to covary within partner, and scores on the same scales were set to covary between partners (e.g., investment for partner 1 was allowed to correlate with investment for partner 2). The error terms for commitment to each partner were also set to covary. The correlation matrix of the variables included in this model is presented in Table 5, and the standardized path coefficients, along with fit statistics for the model, are presented in Table 6. The model had acceptable fit with a Compartive Fit Index (CFI) equal to .96 (a value greater than .95 indicates good model fit) [45].

**Table 5:** Within and between partner correlations of the investment model variables with commitment for each relationship.

	**I**^a^	**S**	**Q**	**C**	***SD***
**I**	.30**^b^^,^^c^	.38**	-.15**	.61**	1.61
**S**	.51**	.12**	-.08**	.62**	1.44
**Q**	-.24**	-.12**	.58**	-.14**	1.65
**C**	.74**	.70**	-.26**	.21**	1.31
***SD***	2.17	1.68	1.74	1.98	

^a^ I = investment, S = relationship satisfaction, Q = quality of alternatives, C = commitment, and SD = standard deviation.

^b^ Correlations for the primary relationship appear above the diagonal line; correlations for the secondary relationship appear below the diagonal. Correlations along the diagonal are between the primary and secondary partners on the same variable.

^c^ **p < .01.

**Table 6:** Within and between partner associations of the investment model variables with commitment for each relationship partner.

Predictors	Outcome Variables	
	Commitment: Primary Partner	Commitment: Secondary Partner
Primary Partner		
Investment	.215**^a^^,^^b^	-.041
Satisfaction	.381**	.019
Quality of Alternatives	**-.019**	.122**
Secondary Partner		
Investment	.003	.497**
Satisfaction	.044**	.472**
Quality of Alternatives	.016	-.182**
R^2^	.47	.68

^a^ Presented in the table are standardized path coefficients. Within partner results are bolded. *n* = 1711. χ2(6) = 106.26, p < .001; CFI = .96.

^b^ ** *p* < .01.

Consistent with Rusbult’s Investment Model [32–33], investment and satisfaction predicted commitment in the expected direction for both primary and secondary relationships, but quality of alternatives only predicted commitment for secondary relationships. The weakest predictor of commitment for each partner was perceived quality of alternatives. The cross-partner paths were comparably smaller in magnitude, but given the large sample size, some of these small coefficients were nonetheless statistically significant and should be interpreted with caution. That said, when individuals reported being more satisfied with their secondary relationship they were more committed to their secondary, and also somewhat more committed to their primary. Further, perceiving greater quality of relationship alternatives for a primary partner was associated with *more* commitment to the secondary partner.

## Discussion

The majority of prior theoretical and empirical work on polyamory has focused on polyamory as part of a general category of CNM, and has compared CNM relationships to monogamous relationships. The present research, using a large community sample, is one of the first to empirically investigate differences specifically in polyamorous individuals’ perceptions of their primary and secondary relationships, the most commonly practiced configuration among polyamorists. We first provide an overall summary of our findings and then discuss the implications of specific findings. We conclude by offering directions for future research.

### Summary of results

Our analyses tested 11 pre-registered hypotheses that can be conceptually grouped into four categories: (1) acceptance and secrecy, (2) investment and commitment processes, (3) relationship communication, and (4) percentage of time spent on sexual activity. Based on our main and exploratory analyses, there is evidence that primary relationships are associated with certain rewards, namely, greater acceptance, less secrecy, higher investment, and commitment levels. There is also a greater amount of communication in primary compared to secondary relationships. However, secondary relationships may offer at least one reward of a newer relationship; percentage of time spent on sexual activity was higher among secondary relationships than primary relationships.

#### Relationship acceptance and secrecy

We conceptualized expressions of acceptance from important others to be one potential reward for primary relationships and the perception of a lack of acceptance to be one cost for secondary relationships. This was suspected, in part, because polyamory is not widely accepted and is a socially stigmatized relationship configuration [22]. Thus, while acceptance from friends and family serves as an important relationship reward, it is unlikely that such acceptance will be afforded to secondary relationships to the same degree as primary relationships given that primary relationships could more easily pass for monogamous relationships. Indeed, some of the strongest and most robust effect sizes in our series of analyses arose from differences in perceived relationship acceptance. Overall, though, levels of acceptance were high for participants in this study and well above the midpoint of the scale, with the exception of family acceptance of secondary partners.

Consistent with differences in acceptance, our results suggest that romantic secrecy is greater with secondary relationships. Although we did not test reasons for relationship secrecy in this study, it is possible they could be reflective of internalized beliefs about how people ought to think or behave. Within a polyamorous relationship, additional relationships beyond the initial dyad may be kept secret to comply with socially accepted norms, which may remain influential even when stigma or lack of acceptance are not actually observed or reinforced. Thus, individuals within polyamorous relationships could choose to maintain their secondary relationships in secrecy, either due to a lack of acceptance from friends and family, or alternatively, secrecy could be a preventative measure to protect against the potential lack of acceptance. Future research is clearly needed to address reasons for romantic secrecy. Future research should also explore the potential costs associated with “coming out” as poly (e.g., problems with one’s family, friends, and career), as well as the potential benefits (e.g., by relieving the stress and burden of concealing a major secret) [37].

#### Relationship investment and commitment processes

Our results suggest that individuals invest more into primary compared to secondary relationships. With regard to investments in romantic relationships, allocation of certain resources (particularly those of a tangible variety, such as money and possessions) is limited in the sense that allocating such resources to one relationship leaves less to be allocated to additional relationships. One implication of this is that investments in a primary relationship may limit the resources available to invest in secondary relationships. Additionally, because secondary relationships are more likely to be socially devalued than primary relationships—as indicated by lower acceptance from friends and family—people in such relationships may invest significantly less in their secondary relationships due to their marginalized nature [34]. Further, or alternatively, because investments usually take time to accrue in a relationship, participants may invest less in secondary relationships simply because those relationships have not existed as long as primary relationships. We tested this possibility in our exploratory analyses, and although difference in relationship length had a significant association with difference in investment, this association did not wholly account for the difference between investment in primary and secondary relationships. Thus, it seems likely that a combination of factors could help account for our finding that investments were lower in secondary compared to primary relationships.

In future research, it would be worth distinguishing among different types of investments (i.e., tangible vs. intangible) in primary and secondary relationships. Tangible investments (e.g., possessions, children) are not easy to distribute equally across relationships, and government-sanctioned marriage typically requires that these investments be tied to a single partner. In light of this, one might predict that primary and secondary relationships would differ when it comes to tangible investments, but not with respect to intangible investments (e.g., time, effort, shared memories), given that the latter are equally available in all relationships [46].

With respect to quality of alternatives in polyamorous relationships and consistent with our prediction, poorer quality of alternatives were reported for primary relationships. However, this was the smallest difference across our series of analyses to emerge. Our exploratory analyses suggest that quality of alternatives is significantly associated with commitment, such that individuals are less committed to partners when they feel they have more alternatives; however, if they feel they have more alternatives to one partner, they feel more committed to the other partner. One caveat to our finding is that it is unclear who our participants were considering as alternatives (e.g., did secondary and other partners “count” as alternatives to the primary relationship? The fact that alternatives for one partner were positively associated with commitment to the other suggests that at least some participants counted their other partners among their alternatives). While we believe that even if participants were considering their other relationships as alternatives, these results are still meaningful and suggestive of the effects quality of alternatives have on consequential relationship phenomena. In future studies that assess quality of alternatives in polyamorous and other CNM relationships, it would be worth using language that more clearly defines what alternatives mean (e.g., including/excluding other partners that one currently has).

Regarding commitment, greater commitment was reported for primary compared to secondary relationships. This result is consistent with previous research findings that marginalization is a significant negative predictor of commitment [34]. Additionally, our exploratory analyses suggest that the individual facets of the Investment model may have some unique associations with commitment.

For example, when individuals reported being more satisfied with their secondary relationship they were more committed to their secondary, and also somewhat more committed to their primary. Additionally, as mentioned above, quality of alternatives was associated with commitment processes in that individuals were more committed to their secondary relationship when they felt they had better alternatives to their primary. It is important to note that our results are specific to the measure of investments, quality of alternatives, and commitment used in this study, which was created and validated on individuals in monogamous relationships.

Work is needed to create and validate measures of commitment on CNM samples–specifically, in terms of the problems with tangible vs. intangible investments and their meaning in polyamorous relationships (discussed earlier), problems with measurement of quality of alternatives (who counts as an alternative?), and about what commitment really means in a polyamorous context. Again, commitment may mean something different in polyamorous relationships and, as such, we may not fully understand the implications. In other words, this finding does not necessarily mean that secondary partnerships are “lesser” or inherently less functional and, due to the issues noted, results should be interpreted with caution.

Taken together, the current results imply that primary relationships are more interdependent than secondary relationships; however, the cross-sectional nature of our data does not allow us to determine whether this equates to greater stability over time with primary compared to secondary relationships. Based upon the existing interdependence literature, one might predict that due to differences in relationship commitment, primary relationships would remain relatively stable, whereas secondary relationships would dissolve more often. Additionally, commitment might mean different things for different relationships. But is this actually the case? This and a number of other interdependence-related questions remain unclear. For instance, when secondary break-ups occur, do new secondary relationships just replace them, leading the same pattern to repeat itself (i.e., primary stability vs. secondary instability)? If so, what is driving this effect—lack of investments, lower satisfaction, greater quality of alternatives, or something else? What are the implications of turnover in secondary relationships for the primary relationship? Does interdependence ebb and flow depending upon the other relationships that one has? Lastly, when a primary relationship does end, do secondary relationships elevate to primary status, or do people seek new primary relationships? How does the secondary partner’s relationship configuration factor into all of this? The current analysis cannot address these questions, but such ideas would be interesting to explore in future studies.

#### Relationship communication

Another reward primary relationships afford is greater communication about the relationship. Not only did survey respondents report greater communication for primary relationships, but when asked to compare the quality of their communication to most people they know, the quality of communication with primary relationship partners exceeded the quality of communication for secondary relationships. This is understandable for several reasons. First, greater communication may be necessary for primary relationships to endure while other relationships are pursued. For example, the decision to communicate about needs and expectations, to negotiate agreements, schedules, and boundaries, and to work through the kinds of problems that emerge when negotiating polyamory, amongst the typical relational problems that can emerge in any relationship, may simply reflect the high level of interdependence that occurs within primary relationships. We would suspect that greater communication is required within primary relationships to successfully navigate not only those relationships, but also relationships amongst other partners. Additionally, one may argue that because participants report a greater relationship duration with primary partners and are more likely to live with primary partners, the greater time communicating—and even better quality of communication—could be an artifact of simply having greater face-to-face access to the primary partners for such communication to occur more easily. However, our exploratory analyses do not support this reasoning. Specifically, the claim that our results speak more to differences between those who are in longer or shorter relationships or those who live together is not supported by the data.

Given different relationship realities of primary-secondary relationships, one question that could better assess the relative importance and role relationship communication has on primary-secondary relationships would be to assess the specific negotiations between these relationships. Future research should explore whether individuals develop different ways of negotiating relationships with primary and secondary partners. While we know primaries experience greater communication, is this because they are better or more practiced at negotiating, or because they are more motivated to negotiate? Furthermore, do more relationships increase the amount of negotiation and communication required or are some people simply better equipped to manage more relationships?

#### Percentage of time spent on sexual activity

One direct reward any relationship can potentially provide is that of sexual activity and the experience of sexual pleasure. As relationships progress, sex and sexuality become key components in most cases. Yet as relationships progress, the amount of sex couples report having also typically declines [39]. One direct reward of secondary relationships, according to our analyses, is the perceived proportion of time spent on sex. Specifically, participants perceive more time spent on sex in secondary compared to primary relationships. However, there are two potential issues with the current conceptualization of time spent on sexual activity. First, the proportion of time spent having sex for primary relationships was 20.74% out of the total amount of time spent with this partner, and the proportion of time spent having sex for secondary relationships was 37.11%, out of the total amount of time spent with this partner. While we asked participants to indicate the percentage of time having sex, we did not ask about the absolute amount of time this involves, or the overall time they spent with their partners in general so that the absolute time could be calculated. It may be the case that partners in secondary relationships are seen less frequently and for less total amount of time and thus more time is spent having sex. With that said, we did assess the proportion of time spent having sex amongst partners who do not cohabitate with either partner. Amongst participants who did not live with either partner, the proportion of time spent having sex in primary relationships increased from 20.74% to 30.02%, an increase of 9.28%, while the proportion of time spent having sex with secondary partners increased from 37.11% to 40.23%, an increase of 3.12% (see Table 3). This suggests that living together largely accounts for the difference in the perceived proportion of time spent having sex, which would make sense intuitively given that individuals who live with their partners would be expected to spend more time together in general (e.g., eating breakfast, reading before bed, etc.). Regardless of this increase, however, significant differences in primary and secondary relationships continued to emerge, though the magnitude of the effect was much smaller, suggesting that cohabitation cannot completely account for the difference in time spent on sexual activity with the primary compared to the secondary, though it does largely account for the difference.

Second, it is hard to know how accurate the estimates for time spent on sex are because we do not know what participants are counting as “sexual activity” (e.g., does spooning and cuddling count? If so, that would likely make the numbers much higher). We cannot assess these possibilities with our current data, although it would be worth exploring in future research. Due to these issues, results should be interpreted with caution.

While the proportion of the time spent having sex was the only reward found for secondary relationships, there may be many other meaningful rewards beyond that which can be attributed to primary relationships. For instance, it is possible that secondary relationships also serve an important role in regard to self-expansion opportunities, given that relationships serve as one of the major sources of self-expansion in our lives [47]. Further, secondary relationships may meet specific needs or desires that primaries are not interested in (e.g., sexual preferences, leisure preferences, etc.). It is also possible that the positive inducements of sexual activity in secondary relationships may have carry-over effects on the primary relationship, either because a partner’s needs that cannot be achieved with primaries are satiated with another and thus not sought after with the primary (leaving both the individual and their partner relieved), or because the sexual expansion with a secondary carries over to the primary. These effects could also be conceptualized as rewards from the secondary relationship in that it benefits the primary. For example, previous research has found that some consensual nonmonogamists report that extradyadic relationships have improved sex within a primary relationship [5, 48, 49, 50]. Hence, future work should explore if, how, why, and when sex within a secondary relationship may improve sex within a primary relationship. Lastly, future work should consider additional rewards—beyond sex—that may be unique to secondary relationships.

## Limitations

Participants for this study were recruited primarily from social media sites frequented by individuals in self-identified polyamorous relationships (e.g., polyamory Facebook groups). While using internet forums and similar data collection methods is common when trying to reach people in marginalized relationships or from marginalized communities, these methods cannot methodologically justify sweeping generalizations. Thus, one major limitation is the source of our sample and, therefore, we urge caution in generalizing the results. Additionally, as this study focuses on a subset of the sample who explicitly identified one partner as primary and another partner as non-primary, future research is needed to assess how partner status (e.g., primary-secondary, co-primary, no primaries) influences the relationships amongst partners in polyamorous relationships.

## Conclusions

This is the first research that has attempted to investigate perceptions of relationships in the context of polyamory. Our results reveal important differences across many theoretically relevant relationship variables in how people perceive primary compared to secondary partners. These differences can help us better understand polyamorous relationships as well as inform future research. The comparisons presented in this manuscript are notable for four reasons: (1) They suggest that individuals are more satisfied with, invested in, and committed to primary relationships, relative to secondary relationships–findings that serve to counter the idea that polyamorous individuals are seeking out alternative relationships due to a lack of satisfaction with the primary; (2) The differences tell us something important about the potential negative effects of the marginalized state of polyamory (e.g., lower acceptance, greater secrecy). People are practicing polyamory, but the stigma against it may be harmful, particularly to secondary relationships; (3) Looking at nuances between relationships also tells us that people may be getting different things out of different relationships, all while maintaining their already established relationships; and (4) Studying CNM relationships is important for testing the boundaries and generalizability of existing relationship models and theory, given that most models/theories of relationships are based on the presumption of monogamy. Polyamory, and CNM relationships more broadly, offer fertile ground for testing the generality of many of these theories and challenging numerous assumptions about relationship processes.
